# Laser-Based Synthesis of TiO_2_-Pt Photocatalysts for Hydrogen Generation

**DOI:** 10.3390/ma15217413

**Published:** 2022-10-22

**Authors:** Elena Fakhrutdinova, Olesia Reutova, Liubov Maliy, Tamara Kharlamova, Olga Vodyankina, Valery Svetlichnyi

**Affiliations:** 1 Laboratory of Advanced Materials and Technology, Tomsk State University, Tomsk 634050, Russia; 2 Laboratory of Catalytic Research, Tomsk State University, Tomsk 634050, Russia

**Keywords:** dark TiO_2_, platinum, defects, laser ablation in liquids, photocatalysis, hydrogen evolution reaction

## Abstract

The development of visible-light active titanium dioxide is one of the key challenges in photocatalysis that stimulates the development of TiO_2_-based composite materials and methods for their synthesis. Here, we report the use of pristine and Pt-modified dark titanium dioxide prepared via pulsed laser ablation in liquid (Nd:YAG laser, 1064 nm, 7 ns) for photocatalytic hydrogen evolution from alcohol aqueous solutions. The structure, textural, optical, photoelectrochemical, and electrochemical properties of the materials are studied by a complex of methods including X-ray diffraction, low-temperature nitrogen adsorption, electrophoretic light scattering, diffuse reflection spectroscopy, photoelectrochemical testing, and electrochemical impedance spectroscopy. Both the thermal treatment effect and the effect of modification with platinum on photocatalytic properties of dark titania materials are studied. Optimal compositions and experimental conditions are selected, and high photocatalytic efficiency of the samples in the hydrogen evolution reaction (apparent quantum yield of H_2_ up to 0.38) is demonstrated when irradiated with soft UV and blue LED, i.e., 375 and 410 nm. The positive effect of low platinum concentrations on the increase in the catalytic activity of dark titania is explained.

## 1. Introduction

Titanium dioxide and materials on the basis thereof are highly efficient and stable photocatalysts for water and air purification from organic pollutants, including volatile organic compounds (VOCs), hydrogen generation and many other applications [1,2,3,4]. In 1972, TiO_2_ became widely known as a hydrogen generation photocatalyst after the publication of Fujishima and Honda [5]. Titanium dioxide is rightfully considered one of the reference materials for photocatalysis. However, it features a number of disadvantages. Thus, the large band gap of ~3.2 eV does not allow the visible range of the spectrum to be used for photocatalysis. This challenge can be solved, e.g., by doping TiO_2_ with metals [6,7,8,9] and nonmetals [10,11], by creating TiO_2_-based composites [12,13].

Despite the rapid development of other photocatalytic materials, e.g., those based on graphene [14] or various types of heterostructures [15], the interest in TiO_2_-based catalysts does not weaken. The development of titania-based catalysts goes in several directions. Of great interest is the so-called dark TiO_2_, a highly defective, Ti^3+^-self-doped titania. For the first time, the dark TiO_2_, active in the visible range of the spectrum, was obtained in 2011 via hydrogenation [16]. Various types of defects in the crystal lattice of titania and their concentration significantly affect the optical, electrical, and photochemical properties determining the catalytic activity of the material [10,17,18,19,20], which stimulates the research on the synthesis and application of dark TiO_2_ in photocatalysis [21,22,23,24,25,26,27,28]. Most methods used to produce defective TiO_2_ are based on the thermal reduction of white TiO_2_ under a vacuum or a strongly reducing atmosphere (H_2_, CO) as well as the bombardment of TiO_2_ with high-energy particles (electrons, argon ions) [29]. High temperature and long processing time lead to particle enlargement. Only the surface of TiO_2_ particles is reduced more efficiently, therefore, the obtained Ti^3+^ sites are easily oxidized in air or dissolved as oxygen in water [30,31]. Therefore, the development of existing methods and the search for new methods to synthesize stable dark TiO_2_ are relevant.

Laser-based methods are also promising ways obtain the dark titania. Two main approaches can be distinguished. In one case, this is laser irradiation of a conventional TiO_2_ powder dispersed in a liquid, which leads to the loss of a part of oxygen [32]. In another case, it is pulsed laser ablation of bulk metallic Ti target in liquid [33]. In the works devoted to the synthesis of titania by pulsed laser ablation in liquid (PLAL), much attention is paid to the study of primary processes during the ablation (study of laser plasma), physical–chemical transformations occurring in the plasma cloud, on the target surface, and in the solution [34,35]. The influence of the energy, time, and spectral parameters of laser radiation [36,37,38] and the nature of the solvent in which the ablation process is carried out [39,40] on the phase composition and dimensional characteristics of TiO_2_ particles are also investigated. At the same time, it is noteworthy that there are practically no works related to the use of dark titanium dioxide powders obtained from the metal with subsequent drying, which is due to the complexity of its production. However, in our research work, we have developed the technology of laser-based synthesis of dark dioxide powders in sufficient quantities for successful application in heterogeneous catalysis [33].

It is noteworthy that pulsed laser ablation in liquid (PLAL) has been increasingly used in recent years to produce catalysts for various purposes [41,42,43], including for the photocatalytic hydrogen generation [44]. Extreme nonequilibrium conditions in laser synthesis (pressure, temperature, ionization, etc.) stimulate physical–chemical processes leading to the formation of metastable phases, defective structures and composites, the formation of which by other methods is rather difficult, or even impossible [41,42].

In Refs. [45,46], we obtained dark titania by nanosecond PLAL in distilled water. It features an intense absorption in the visible region of the spectrum caused by the presence of defects of various nature, and exhibits increased photocatalytic activity in the decomposition of the dye Rhodamine B and phenol, as well as an antibacterial effect against *S. Aureus* and *E. Coli* [45].

In the present work, we used PLAL in water to obtain and optimize dark titania for photocatalytic hydrogen evolution reaction (HER). The photocatalytic efficiency in HER was significantly increased by modifying the dark titania with small concentrations of platinum also obtained by PLAL in water.

## 2. Materials and Methods

### 2.1. Sample Preparation

Pure dark titania was obtained by PLAL of metallic Ti target (99.5% purity) in distilled water. The focused Nd:YAG laser radiation (LOTIS TII, model LS2131M-20) with a wavelength of 1064 nm, pulse energy of up to 180 mJ, pulse repetition duration and frequency of 7 ns and 20 Hz, respectively, was used. The laser power density on the target surface was ~0.5 GW/cm^2^. The colloidal solution synthesized by PLAL was dried in air (the sample was designated as “Ti_ini”). The powder obtained during drying was annealed in a muffle furnace in air in the temperature range of 250–800 °C. The resulting series of samples was designated as “Ti_x”, where x was the annealing temperature.

Platinum-modified dark titania (TiO_2_-Pt) was obtained as follows. First, colloidal solutions of titanium dioxide nanoparticles (as described above) and platinum were separately obtained by the PLAL. A metallic Pt target (99.99% purity) was used for PLAL of Pt. Platinum ablation was carried out in ethanol (96%) with the same laser at a focused laser power density of ~0.4 GW/cm^2^. Then, the freshly prepared colloidal solutions were rapidly mixed and processed in an ultrasonic bath. Then, the mixture of colloidal solutions was also dried in air at a temperature of 60 °C and annealed in air at 400 °C. The platinum content in the samples varied from 0.1 to up to 2 wt.%. The resulting series of samples was designated as “yPt/Ti_400”, where y was the mass content (%) of platinum.

### 2.2. Material Characterization

The crystal structure of the samples was studied by X-ray diffraction (XRD) using the XRD-7000 diffractometer (Shimadzu, Japan) with a monochromatic CuKα radiation (1.54 Å) in the 2θ angle range of 20–90° and a scanning speed of 0.02°/s. The phase composition was analyzed using the PDF-4 database (Release 2021 RDB). To refine the lattice parameters and determine the coherent scattering regions (CSR) for crystal phases, the POWDER CELL 2.4 full-profile analysis program was used.

The low-temperature nitrogen adsorption was studied on the TriStar II 3020 equipment (Micromeritics, Norcross, GA, USA). Prior to the experiments, the samples were degassed either at 150 °C in a vacuum (10^−2^ Torr) for 2 h using a laboratory degassing station, or with the VacPrep Degasser (Micromeritics, USA). The Ti_ini sample was degassed at a temperature of 25 °C. The specific surface area was determined using the Brunauer–Emmett–Teller (BET) method.

The electrokinetic properties of the samples were studied by the electrophoretic light scattering using the phase analysis light scattering (PALS) technique on the Omni S/N (Brookhaven, NY, USA) analyzer equipped with the BI-ZTU autotitrator (Brookhaven, NY, USA). The samples were dispersed in distilled water at a concentration of 25 mg/L using ultrasound. The concentration of the Pt dispersion prepared by PLAL and used as a reference sample was 15 mg/L. To determine the pH of the isoelectric point (IEP), the colloids were titrated using the HNO_3_ solution.

The absorption of the powders was studied by the diffuse reflection spectroscopy (DRS) using the Cary 100SCAN spectrophotometer (Varian, Belrose, NSW, Australia) with the addon DRA-CA-30I (Labsphere, North Sutton, NH, USA) in the wavelength range of 230–800 nm. MgO was used as a measurement standard. The width of the band gap was estimated by the Tauc method for the indirect band gap transition [47].

The electrochemical experiments were carried out using the CHI 660E electrochemical workstation (CH Instruments, Bee Cave, TX, USA) with a conventional three-electrode system. The working electrode was a TiO_2_-based photocatalyst drop-casted onto the pre-cleaned fluorine-doped tin oxide (FTO) glass (<10 ohm/sq) and sintered at 200 °C for 2 h. The graphite rod and Ag/AgCl(1 M KCl) electrode were used as a counter electrode and reference electrode, respectively. A working solution was purged with argon prior to each electrochemical experiment to remove dissolved oxygen. An aqueous solution of 0.1 M Na_2_SO_4_ with an addition of 5 wt.% glycerol served as a working electrolyte for photoelectrochemical (PEC) tests. The transient photocurrent tests were carried out at an open circuit potential under chopped LED irradiation with the off/on cycle of 10 s under ambient conditions. A 3 mW LED (λ = 375 nm) served as a soft UV light irradiation source. Electrochemical impedance spectroscopy (EIS) measurements were carried out in 0.1 M KNO_3_ electrolyte containing 5 mM ferro/ferricyanide redox couple. The EIS measurements were carried out at the frequency from 1 MHz to up to 1 Hz with the amplitude of 10 mV at the open circuit potential of 0.2 V. The Mott–Schottky dependencies were recorded in the dark in 0.1 M Na_2_SO_4_ electrolyte at a frequency of 1 kHz with an amplitude of 10 mV in a potential range from −0.5 to 1.5 V. Potentials vs. Ag/AgCl (1 M KCl) were converted into potentials vs. reversible hydrogen electrode (RHE) according to the Nernst equation: E_RHE_ = E_Ag/AgCl_ + 0.059pH + 0.222.

### 2.3. Studies of Photocatalytic HER Activity

**Figure 1:**
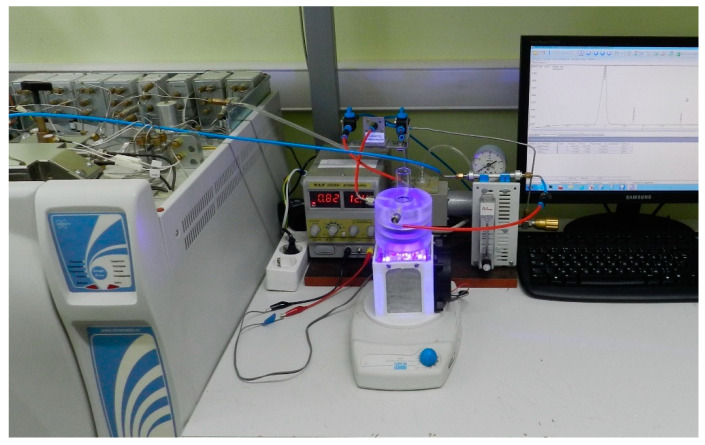
Image of the photocatalytic unit for hydrogen generation with online chromatographic detection.

The photocatalytic properties of the materials were investigated in the hydrogen evolution reaction (HER). The experiment was carried out in a closed gas system equipped with a cylindrical quartz reactor using argon as a carrier gas. Prior to the experiment, 50 mg of the photocatalyst was dispersed for 10 min in 100 mL of a sacrificial reagent solution. During the experiment, the catalyst was continuously stirred using a magnetic stirrer (rpm = 1100 rpm). To prevent the formation of a funnel during the mixing, a cylindrical flask was placed inside the reactor. The radiation source was 16 LED with wavelengths of 375 nm (total radiated power was 0.3 W) or 410 nm (total radiated power was 1.75 W). The LED evenly irradiated the outer side surface of the reactor. The experiments were carried out at room temperature (22 ± 2 °C). To prevent heating and exclude thermophotocatalysis, the LEDs were mounted on aluminum radiators and blown with air using fans. Figure 1 shows the image of the reactor part of the unit.

The amount of hydrogen formed was determined using the Crystal 5000 gas chromatograph (Chromatek, Yoshkar-Ola, Russia) equipped with a thermal conductivity detector (TCD). Prior to photocatalysis, the suspensions were purged with argon to minimize nitrogen and oxygen contents in the system.

## 3. Results and Discussion

### 3.1. Structure and Optical Properties of Samples

Table 1 shows the characteristics of the obtained dark titania samples in comparison with those of commercial Hombifine TiO_2_.

The initial Ti_ini sample is X-ray amorphous and features no long-range order. Higher content of amorphous phase is characteristic of the preparation of semiconducting oxides during ablation in liquid, especially ablation of hard-melting materials [48,49,50]. The crystal structure begins to form during the annealing at ~300 °C. After annealing at 400 °C, a structure consisting of 90% and 10% of anatase and rutile phases, respectively, is formed in the sample. With an increase in the annealing temperature, the proportion of rutile increases, but even at 800 °C, a complete phase transition of anatase/rutile does not occur. The initial sample features a high specific surface area of 227 m^2^/g that decreases as the calcination temperature increases.

**Figure 2:**
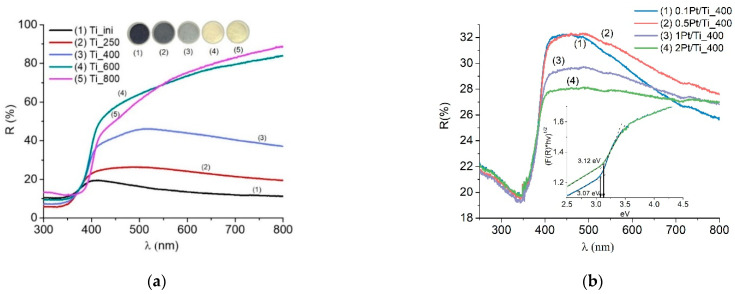
UV-Vis DRS of TiO_2_ (**a**) and TiO_2_-Pt (**b**) powders.

Figure 2a shows the diffuse reflection spectra and images of the samples annealed at different temperatures. Figure 2a shows that samples with a processing temperature of up to 400 °C feature an intense absorption in the entire visible range that is connected to the presence of defects of various nature (different types of oxygen vacancies, Ti^3+^ ions, self-trapped excitons) [45]. Upon annealing at 600 °C and above, the defect of the samples decreases significantly, which is confirmed, among other things, by the luminescence studies [45,46]; the absorption in the visible region decreases. The band gap width of the samples, estimated according to the Tauc method, is about ~3 eV and remains practically unchanged.

Based on the above, the samples modified with platinum (Figure 2b) were all annealed at a temperature of 400 °C, since such samples possess a crystalline structure. Anatase, which retains a relatively high specific surface area and absorption in the visible spectral region (Figure 2a), i.e., can be attributed to dark titania. It is noteworthy that Ti_400 demonstrates the highest HER efficiency, as will be shown below.

According to the XRD data (Table 1, Figure S1), the introduction of platinum initiates the appearance of the third phase of titanium dioxide, namely brookite. The increase in the content of the brookite phase occurs due to a decrease in the proportion of anatase and correlates with an increase in the platinum content in the samples. In the XRD patterns, platinum is detected at concentrations from 0.5 wt.% (a reflection at 2θ of 39° is the (111) plane of the metallic Pt with a cubic syngony).

The HR TEM and XPS data that we obtained earlier [33] show that even for the samples with a platinum content of 2 wt.%, platinum is rather evenly distributed in the titania matrix at the micro level, without the formation of large agglomerates. Small platinum particles have an average size of ~2 nm with insignificant content of larger particles with sizes up to 10 nm. In this case, platinum is present in the samples both in the form of the metal phase Pt^0^ and in the form of Pt^4+^, and the effective metal support interaction (MSI) occurs between the Pt and defective TiO_2_ [33].

The DRS results show that the platinum introduction practically does not affect the position of the edge of the absorption band. The band gap width does not change within the error provided according to the Tauc method for the dark samples, since the particle size and phase composition practically do not change in the samples (the predominance of anatase modification). Therefore, the introduction of a small amount of platinum will not have a special effect on the position of the edge of the absorption band. Ionic platinum can feature additional levels in the TiO_2_ band gap, which is associated with the appearance of additional absorption in the visible and near-IR spectral regions, which increases as the Pt content increases. Absorption in the visible and near-IR spectral regions is expected to increase upon the addition of platinum.

Figure S2 and Table 2 show the results of the study of electrokinetic characteristics for aqueous colloids of platinum samples annealed at 400 °C, which were further studied in the photocatalytic HER. The zeta potential of the particles in the suspension (ζ_o_) of the Ti_400 sample is −13 mV at pH = 7.2 (pH_o_). Titration of the initial suspension with HNO_3_ solution is accompanied by an increase in the zeta potential of the particles with a change in the charge sign at pH = 4.7, which can be attributed to the TiO_2-δ_ surface characterized by the formation of Ti–O^–^/Ti–OH equilibria during the titration. A relatively low pH value of the isoelectric point (IEP) for TiO_2__400 compared to those values characteristic of anatase and rutile can be associated with a high defect structure of the resulting PLAL material.

The zeta potential of the particles in the Pt suspension is −38 mV at pH = 4.7. The negative zeta potential of the particles obtained by PLAL is due to the unique chemical properties of their surface. During the ablation, some of the atoms on the surface of the obtained noble metals are oxidized to Me^n+^ and can interact with water and CO_2_ dissolved in it to form Me–O^–^ and Me–CO_3_^–^, generating a negatively charged surface layer of particles [51,52]. Titration of the initial suspension with HNO_3_ solution is accompanied by an increase in the zeta potential of the particles with a change in the charge sign at pH = 2.1.

The dependences of the zeta potential of the particles on the pH of the suspension for yPt/Ti_400 samples, y = 0.1 … 1, lie between the dependences obtained for Ti_400 and Pt samples, smoothly shifting to the lower pH region. This is due to the additivity of the zeta potential of multiphase particles. Data for a high Pt concentration fall out of the series, which may indicate the beginning of the agglomeration of Pt particles and lower uniformity of the sample.

### 3.2. Photocatalytic Activity of Titania Prepared by PLAL

**Figure 3:**
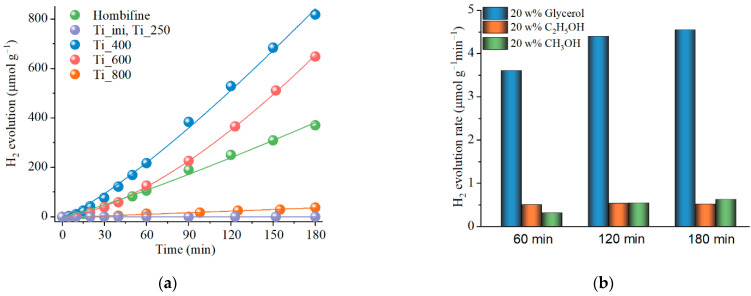
(**a**) HER in the presence of different Ti_x samples (glycerol, as a sacrificial agent); (**b**) the H_2_ evolution rate for Ti_400 in the presence of various sacrificial agents under LED 375 nm source.

Figure 3a shows the kinetic dependences for photocatalytic hydrogen evolution in the presence of a number of Ti_x catalysts annealed at different temperatures in comparison with the commercial Hombifine TiO_2_ catalyst. When an aqueous solution of the sacrificial agent is irradiated without a catalyst, hydrogen is not released. Hydrogen is also not released in the presence of Ti_ini and Ti_250 samples, while these materials show relatively high activity in the decomposition of model phenol compound when irradiated with a metal halide 70 W lamp [45]. With an increase in the annealing temperature, when the samples become crystalline, the process of photocatalytic hydrogen release begins. The largest amount of hydrogen is released in the presence of the Ti_400 sample, namely, 0.82 mmol/g for 3 h of irradiation, which is ~2.2 times higher than for the Hombifine TiO_2_. A further increase in the annealing temperature leads to a decrease in the efficiency of hydrogen evolution. Thus, the hydrogen evolution decreases by ~10% for the Ti_600 sample and is not practically observed for the Ti_800 sample. This is associated with an increase in the fraction of rutile in the samples (the Ti_800 sample contains 58% rutile in its composition), a reduction in the number of defects that are active sites in the TiO_2_ system, an increase in crystallite size, and a decrease in the specific surface area (Table 1). Therefore, the results obtained indicate that the crystal structure and dispersion of the titania materials after thermal treatment strongly affect their photocatalytic activity in HER, with the best activity being observed for the Ti_400 sample primarily formed by dispersed titania with defective anatase crystal stricture. Detailed studies on the effect of experimental parameters on the HER efficiency, as well as experiments for a series with platinum, were further carried out for the samples annealed at 400 °C.

The dependences of the efficiency of hydrogen evolution at different catalyst loadings (Figure S3) as well as the nature of the sacrificial agent (Figure 3b) were investigated. It was found that the optimal loading for this system was 0.5 g/L. A further increase in mass did not lead to an increase in the amount of hydrogen released. Testing of sacrificial reagents, i.e., alcohols preferred for oxide photocatalysts [53], from three common additives (methanol, ethanol, and glycerol) was performed. The experiment was carried out for 20 wt.% of sacrificial agents in aqueous solutions without any additional additives. The pH of the medium remained relatively neutral (pH 6.2–6.9). The rates of hydrogen evolution in the presence of monatomic alcohols were similar and practically did not change with time. The rate of hydrogen evolution in triatomic alcohol (glycerol) was ~seven times higher than the one in methanol and ethanol and increased slightly over time. Such a difference in the rates of hydrogen evolution can be explained by the fact that one of the main intermediate compounds for alcohols with a short carbon chain is carbon monoxide (CO), which, being adsorbed on the active sites, can limit further alcohol adsorption on the photocatalyst surface, thereby slowing down the hydrogen evolution rate [54]. In turn, in polyatomic alcohols (such as glycerol), the binding of photogenerated holes occurs faster [55,56], and a large number of OH groups can serve as an anchor for the chemisorption of alcohols on the photocatalyst surface [57]. Thus, glycerol is more suitable for use as a sacrificial reagent for the studied materials.

### 3.3. Effect of Pt Addition on the Photocatalytic Activity of Titania Obtained by PLAL

**Figure 4:**
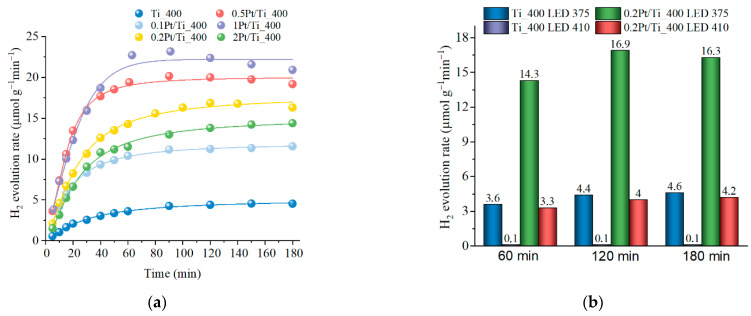
The H_2_ evolution for (**a**) yPt/Ti_400 sample under irradiation by LED 375 nm; (**b**) LED 375 nm and LED 410 nm irradiation.

It is known that the use of Pt as a co-catalyst can provide active sites for the reduction/oxidation reaction, increase absorption in the visible region, and also promote charge separation due to the formation of the Schottky transition between a semiconductor and a metal [58]. Figure 4 and Table 3 show the results of a study of the photocatalytic properties of Pt-activated dark titania samples obtained by PLAL.

Figure 4a shows data on the rate of photocatalytic hydrogen evolution for samples with different Pt contents when irradiated with the LED with a wavelength of 375 nm. The Pt addition in the amount of only 0.1 wt.% provides a 2.5-time increase in the rate of hydrogen evolution compared to a similar sample without platinum (Ti_400). With further increase in the Pt addition to up to 1 wt.%, the rate of hydrogen evolution continues to increase, but not as efficiently. When the Pt content increases to up to 2 wt.%, the rate of hydrogen evolution begins to decrease, which is consistent with the literature data [59,60] as well as with the electrokinetic measurements (Figure S2). Excessive loading can lead to aggregation of Pt nanoparticles on the TiO_2_ surface, as well as blocking of access to the active surface sites, e.g., oxygen/Ti^3+^ vacancies, which reduces the efficiency of not only the photocatalytic processes, but also, e.g., the formaldehyde oxidation [61] and CO conversion [62]. High concentrations of Pt^4+^ can increase the recombination rate, which leads to a decrease in photocatalytic activity.

In addition to UV LED, an experiment was conducted to generate hydrogen by irradiating blue LED with a wavelength of 410 nm (Figure 4b). The quantum energy of 410 nm is lower than the bandgap width, and the excitation can proceed through the defective states, including those formed during the Pt doping. The use of blue LED leads to a decrease in the rate of hydrogen evolution (Figure 4b). In the case of an undoped Ti_400 sample, the rate of hydrogen evolution is significantly reduced (~36 times), and in the case of a sample with platinum (0.2Pt/Ti_400) it decreases only ~four times.

To correctly compare the efficiency of HER with different irradiation that takes into account the radiation power of diodes and the fraction of energy absorbed in the medium, we measured these parameters and calculated the apparent quantum yield (AQY):

AQY = N(H_2_)/N(hv), (1)

where N(H_2_) was the number of evolved H_2_ molecules and N(hv) was the number of incident photons.

For the excitement at 375 nm LED, the AQY increases by an order of magnitude for Pt-modified samples in comparison with the unmodified Ti_400 and Hombifine TiO_2_ and reaches 0.38 for the 1Pt/Ti_400 sample. When using blue LED, the AQY for the 0.2Pt/Ti_400 sample decreases ~30 times, while for the undoped Ti_400, it is reduced by six orders of magnitude.

**Figure 5:**
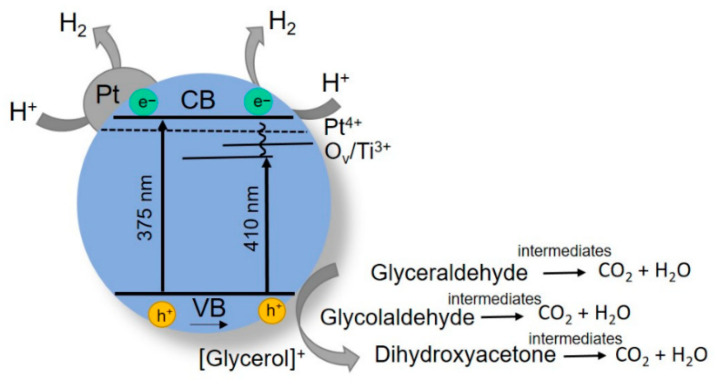
Scheme of photoprocesses for HER in presence of defective Pt/TiO_2_ catalyst.

Summarizing the results for dark titania modification with platinum, Figure 5 shows a scheme of photoprocesses occurring during HER on Pt-TiO_2_ in the presence of glycerol as a sacrificial agent. The oxygen/Ti^3+^ vacancy levels are shown in the bandgap of the TiO_2_ particle along with those of ionic Pt^4+^, which are responsible for absorption in the visible region of the spectrum. The presence of defects increases the density of donors due to the electron capture, which can significantly enhance the transfer and separation of charges [63], as well as shifting the Fermi level of TiO_2_ towards the conduction band [64]. Black arrows in the scheme show electron transfer when using LED 375 nm (excitation of the edge of the absorption band) and LED 410 nm (electron transfer through defective levels in the energy structure). Gray arrows show the hydrogen release without and with the participation of Pt, as well as the interaction of holes with glycerol, followed by its decomposition into various products, taking into account the decomposition on various TiO_2_ modifications [65].

### 3.4. Electrochemical Analysis of the Photocatalytic Activity of Dark Pt/TiO_2_ Obtained by PLAL

**Figure 6:**
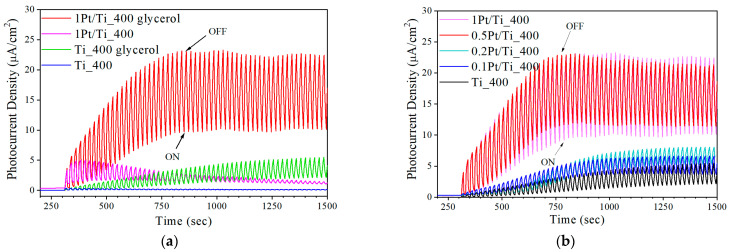
Transient photocurrent responses for pristine and Pt-doped TiO_2_ in 0.1 M Na_2_SO_4_ electrolyte: the effect of (**a**) glycerol addition to the electrolyte and (**b**) Pt loading.

To confirm the mechanisms of changes in the photocatalytic properties of dark titania samples, the data from the electrochemical studies of the samples were used. Photoelectrochemical (PEC) properties of obtained photocatalysts were characterized by the transient photocurrent response in order to assess the material possibility of the electron production and charge transfer processes under illumination. The PEC tests were conducted in a Na_2_SO_4_ electrolyte with an addition of the glycerol serving as a sacrificial agent to improve the charge separation and increase photocurrent. The photocurrent curves (Figure 6) showed that the electron–hole pairs were effectively produced under LED 375 nm illumination by the materials under study. The samples showed rapid, uniform, and stable photocurrent responses, suggesting good stability of the composites under illumination in the solution.

As Figure 6b shows, the modification of TiO_2_ with Pt nanoparticles led to an increase in photocurrent density. The enhanced photocurrent indicated the enlargement of the photogenerated carrier transport rate and a decrease in the photogenerated charge carrier recombination rate. This occurred due to the enhancement of the electron transfer and charge separation by the Pt nanoparticles. The photocurrent density for the Pt/TiO_2_ photocatalysts increased with an increase in the Pt content from 0.1 wt.% to 0.5 wt.%, indicating that the photocatalytic activity also increased. With a further increase in the Pt content, the photocurrent did not increase, which is consistent with the data of photocatalytic studies. The results of the photocurrent investigation are consistent with the EIS data.

**Figure 7:**
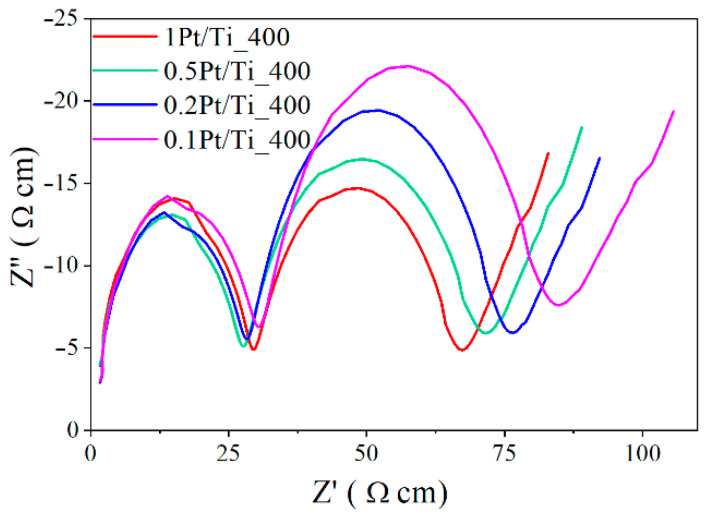
EIS Nyquist plots of the Pt-doped TiO_2_-based photocatalysts; electrolyte solution is 0.1 M KNO_3_ containing 5 mM [Fe(CN)_6_]^3−/4−^.

The EIS with the use of ferro/ferricyanide redox couple have confirmed the electrocatalytic activity of the Pt supported on TiO_2_ (Figure 7). The radius of the arc on the EIS Nyquist plot is related to the electron transfer resistance at the photocatalyst surface. The electron transfer resistance of Pt-doped TiO_2_ decreases in the raw of Pt loading from 0.1 wt.% to 1 wt.%, indicating an enhancement in the electron transfer and charge separation.

The photocatalytic properties of a semiconductor photocatalyst are closely connected to its electronic energy level positions (the Flat band potential). The position of the energy bands was estimated by the Mott–Schottky analysis. The Mott–Schottky plot shows a relation between the apparent capacitance 1/C^2^ and applied potential in the depletion region across a semiconductor–electrolyte junction (2):

(2) $$\frac{1}{C_{sc}^{2}}=\frac{2}{e\varepsilon \varepsilon_{0}N_{d}}\left(E-E_{fb}-\frac{kT}{e}\right)$$

where *C_sc_* is the capacitance of the space charge region in the semiconductor, *e* is the elementary charge, *ε* is the dielectric constant of the semiconductor, *ε*_0_ is the permittivity of free space, *N_d_* is the carrier density, *E* is the applied potential, *E_fb_* is the flat band potential, *k* is the Boltzmann constant, and *T* is the absolute temperature.

According to the equation, the extrapolation of the linear part of the Mott–Schottky plot gives the flat band potential value, and the slope is related to the carrier density of a semiconductor. As expected, the Mott–Schottky plots of the TiO_2_-based samples showed a positive slope in the linear region in accordance with their n-type nature (Figure S4); thus, the *E_fb_* values relate to the conduction band potential. The *E_fb_* values were found to be 0.24 and 0.15 V vs. RHE for Ti_400 and 1Pt/Ti_400, respectively. The electron density was found to be 3.68·10^21^ and 6.53·10^21^ carriers cm^−3^ for Ti_400 and 1Pt/Ti_400, respectively. Doping of TiO_2_ with Pt nanoparticles resulted in a negative shift of the conduction band and an increase in the dopant density due to the electrocatalytic effect of platinum and improved electron transfer.

Thus, the results of the electrochemical studies explain the Pt effect on the photocatalytic activity of dark titania.

## 4. Conclusions

In summary, pulsed laser ablation in a liquid makes it possible to obtain pure and highly defective dark titania that intensively absorbs light in the whole visible range. The crystal structure and dispersion of the titania-based materials after thermal treatment was shown to strongly affect their photocatalytic activity in hydrogen evolution from alcohol, primarily glycerol, aqueous solutions under LED sources of 375 and 410 nm. The best activity was observed for the sample annealed at 400 °C, which was primarily formed by dispersed titania with defective anatase crystal stricture. In terms of hydrogen generation efficiency, it was superior to the commercial Hombifine TiO_2_ catalyst. 

Glycerol was shown to be more suitable for use as a sacrificial reagent for the studied materials, while methanol and ethanol reduced the hydrogen evolution rate.

Modification of dark titania with platinum in low concentrations (up to 1 wt.%) results in further improvement in the photocatalytic activity of the materials, with the rate of hydrogen evolution being increased up to ~five times. The maximum apparent quantum yield of hydrogen generation was 0.38 under a LED source of 375 nm. When irradiated with the blue LED source of 410 nm, despite the decrease in the HER quantum efficiency, hydrogen generation continued that indicated the effective operation of Pt and structural defects.

The positive effect of low platinum concentrations on the increase in the photocatalytic activity of dark titania was explained on the basis of electrochemical studies. An increase in activity was consistent with the increase in the density of photogenerated charge carriers, as well as a decrease in the electron transfer resistance, i.e., the Pt introduction made the process of photogeneration of charge carriers more efficient.

Further development of research to create highly efficient photocatalysts based on dark titanium dioxide for hydrogen generation will be focused on controlling the size and state of platinum during the doping, as well as the creation of heterostructures of dark titanium dioxide (n-type semiconductor) with metal oxides (Cu, Co) with p-type conductivity using PLAL technology.

## Figures and Tables

**Table 1:** Phase content, crystallite size, band gap value, specific surface area of PLAL samples.

Sample	Phase Content Titania (%)			CSR Size (nm)	Band Gap (eV)	S_BET_ (m^2^/g)
	Anatase	Rutile	Brookite			
Hombifine	95	5	–	15	3.03	296
Ti_ini	–	–	–	–	3.06	227
Ti_250	–	–	–	–	3.06	170
Ti_400	90	10	–	24	3.03	86
Ti_600	66	34	–	38	3.01	50
Ti_800	42	58	–	105	3.00	7
0.1Pt/Ti_400	80	12	8	24	3.07	108
0.2Pt/Ti_400	80	12	8	23	3.07	94
0.5Pt/Ti_400	80	12	8	24	3.07	95
1Pt/Ti_400	75	12	13	23	3.09	95
2Pt/Ti_400	71	12	17	25	3.12	90

**Table 2:** Electrokinetic parameters of suspensions of PLAL samples.

Sample	pH_o_	ζ_o_, mV	pH_IEP_
Ti_400	7.2	–13	4.7
0.1Pt/Ti_400	5.3	–15	3.6
0.2Pt/Ti_400	5.7	–20	3.4
0.5Pt/Ti_400	5.3	–19	2.6
1Pt/Ti_400	5.2	–12	4.1
2Pt/Ti_400	7.2	–13	4.7
Pt	4.7	–38	2.1

**Table 3:** Hydrogen evolution and apparent quantum yield for 3 h of irradiation.

Sample	LED 375		LED 410	
	HER (mmol/g)	AQY	HER (mmol/g)	AQY
Hombifine	0.37	0.04	–	–
Ti_400	0.82	0.08	0.02	~10^−8^
0.1Pt/Ti_400	2.08	0.21	–	–
0.2Pt/Ti_400	2.94	0.29	0.76	0.01
0.5Pt/Ti_400	3.45	0.34	–	–
1Pt/Ti_400	3.77	0.38	–	–
2Pt/Ti_400	2.59	0.26	–	–

## Data Availability

The data presented in this study are available on request from the corresponding authors.

## Supplementary Materials

The following supporting information can be downloaded at: https://www.mdpi.com/article/10.3390/ma15217413/s1, Figure S1. XRD pattern of dark titania samples doped with platinum. Figure S2. Dependence of the zeta potential of the particles on the pH of the suspension. Figure S3. The effect of the catalyst loading for Ti_400 and glycerol as a sacrificial agent. Figure S4. Mott−Schottky plots for Ti_400 and 1Pt/Ti_400 obtained in 0.1 M Na_2_SO_4_ electrolyte (pH 6.42).
