# Supplementary material for: Laser-Based Synthesis of TiO2-Pt Photocatalysts for Hydrogen Generation
Source: Materials (Basel). 2022 Oct 22;15(21):7413. doi: 10.3390/ma15217413 (PMC9655175; doi:10.3390/ma15217413)
Supplement: Supplementary file 1 [file materials-15-07413-s001.zip › materials-1985683-supplementary.pdf]

materials

Supplementary Materials

Laser-based Synthesis of TiO_2-Pt Photocatalysts for Hydrogen Generation

Elena Fakhrutdinova[1]*, Olesia Reutova[1], Liubov Maliy[2], Tamara Kharlamova[2], Olga Vodyankina[2], Valery Svetlichnyi[1]*

[1] Laboratory of Advanced Materials and Technology, Tomsk State University, Tomsk 634050, Russia
[2] Laboratory of Catalytic Research, Tomsk State University, Tomsk 634050, Russia
* Correspondence: fakhrutdinovaed@gmail.com (E.F.); v_svetlichnyi@bk.ru (V.S.)

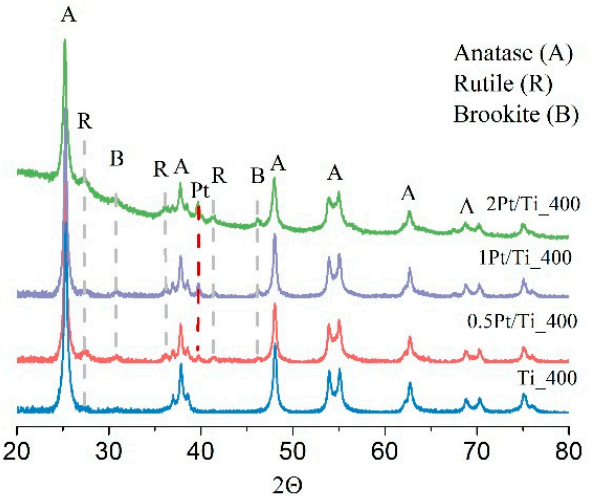

Figure S1. XRD pattern of dark titania samples doped with platinum.

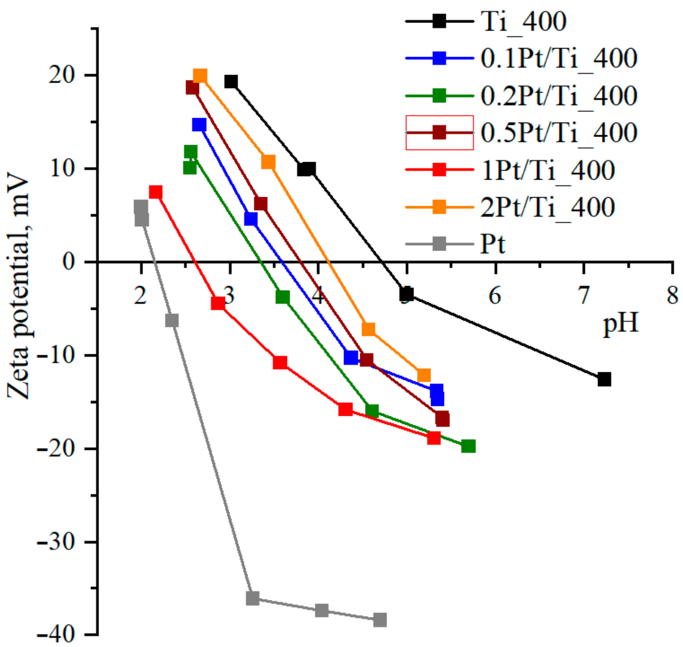

Figure S2. Dependence of the zeta potential of the particles on the pH of the suspension.

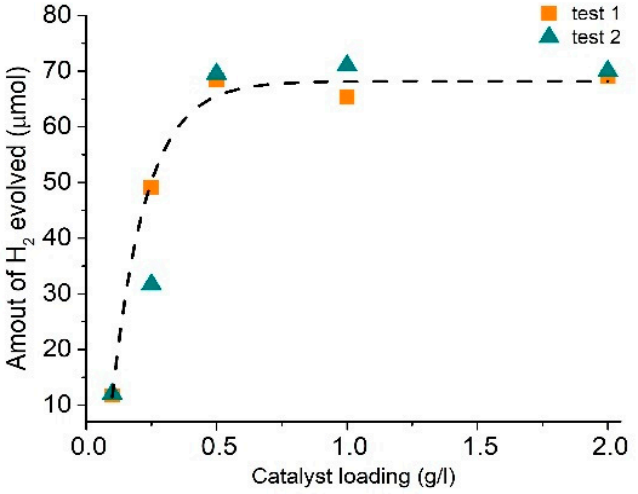

Figure S3. The effect of the catalyst loading for Ti_400 and glycerol as a sacrificial agent.

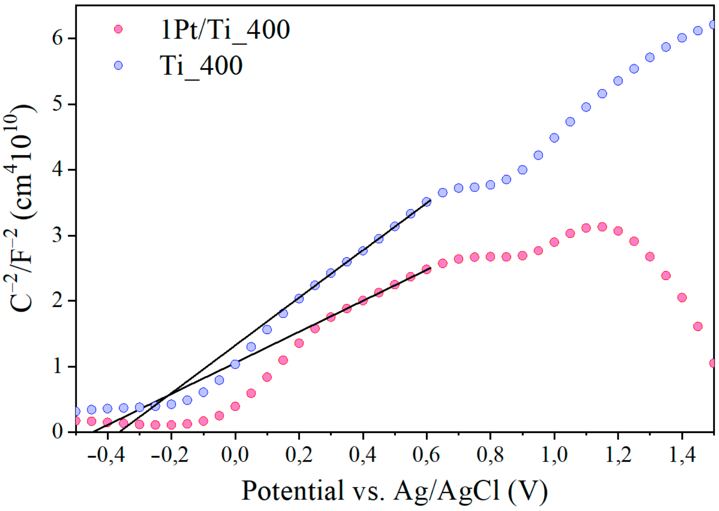

Figure S4. Mott–Schottky plots for Ti_400 and 1Pt/Ti_400 obtained in 0.1 M Na_2SO_4 electrolyte (pH 6.42).
